# Entrapment of Glucose Oxidase and Catalase in Silica–Calcium–Alginate Hydrogel Reduces the Release of Gluconic Acid in Must

**DOI:** 10.3390/gels9080622

**Published:** 2023-08-01

**Authors:** David del-Bosque, Josefina Vila-Crespo, Violeta Ruipérez, Encarnación Fernández-Fernández, José Manuel Rodríguez-Nogales

**Affiliations:** 1 Área de Tecnología de los Alimentos, Escuela Técnica Superior de Ingenierías Agrarias, Universidad de Valladolid, 34004 Palencia, Spain; d.bosque@csic.es (D.d.-B.); encarnacion.fernandez@uva.es (E.F.-F.); 2 Área de Microbiología, Escuela Técnica Superior de Ingenierías Agrarias, Universidad de Valladolid, 34004 Palencia, Spain; josefinamaria.vila@uva.es (J.V.-C.); violeta.ruiperez@uva.es (V.R.)

**Keywords:** acidity, hybrid capsule, organic-inorganic gel, siliceous material, sol–gel network

## Abstract

Glucose oxidase (GOX) and catalase (CAT) were co-immobilized in silica–calcium–alginate hydrogels to degrade must glucose. The effect of the enzyme dose (1.2–2.4 U/mL), the initial must pH (3.6–4.0), and the incubation temperature (10–20 °C) on the glucose consumption, gluconic acid concentration, pH, and color intensity of Verdejo must was studied by using a Box–Behnken experimental design and comparing free and co-immobilized enzymes. A reduction of up to 37.3 g/L of glucose was observed in co-immobilized enzyme-treated must, corresponding to a decrease in its potential alcohol strength of 2.0% vol. (*v*/*v*), while achieving a slight decrease in its pH (between 0.28 and 0.60). This slight acidification was due to a significant reduction in the estimated gluconic acid found in the must (up to 73.7%), likely due to its accumulation inside the capsules. Regarding the operational stability of immobilized enzymes, a gradual reduction in glucose consumption was observed over eight consecutive cycles. Finally, co-immobilized enzymes showed enhanced efficiency over a reaction period of 48 h, with an 87.1% higher ratio of glucose consumed per enzyme dose in the second 24 h period compared with free enzymes. These findings provide valuable insights into the performance of GOX–CAT co-immobilized to produce reduced-alcohol wines, mitigating excessive must acidification.

## 1. Introduction

Global warming is no longer a distant threat but a reality whose present and future consequences must be addressed in order to safeguard the quality of the wines of today’s wine-growing areas [1,2,3]. Rising average temperatures and changing weather conditions alter the normal cycle of the vines [3] and grapes [4] and generate imbalances in the composition and concentration of the musts, disrupting the production of quality wines. The variability of meteorological phenomena and the increase in temperature forces each producer to adapt to the changes by choosing the strategy that best suits their winemaking needs. Among the pernicious effects of the increase in temperature is mainly a greater mismatch between phenolic and technological maturation in the grapes [5], generating musts with a high accumulation of sugars and lower acidity [6]. This leads to wines with a higher alcohol content and an altered sensory profile [7]. In addition, the alteration of each of these parameters by itself can have multiple other counterproductive repercussions, following a chain reaction, both in the different stages of winemaking process and in the final quality of the wine [8]. For example, a high concentration of sugars can cause significant osmotic stress on the yeast population, leading to sluggish or stuck fermentation, or a high pH value can lead to microbial instability, off-flavors, and color changes [9]. Therefore, to find the least possible impact on the final organoleptic quality of the wine, new strategies are being developed to compensate and readjust the altered parameters of the sugar concentration and pH of the musts and/or the alcohol content of the resulting wines [10,11,12]. These strategies incorporate new techniques and technologies, both physico-chemical and biological [13], with different levels of complexity and cost [14]. They can range from (i) changes and management in viticulture [15] to winery processes during (ii) pre-fermentation, (iii) fermentation, and (iv) post-fermentation. 

An alternative biotechnological strategy based on the use of enzymes involves the treatment of must with glucose oxidase (GOX) and catalase (CAT) to reduce its glucose concentration before the onset of alcoholic fermentation, leading to the production of wines with a reduced alcohol content. GOX is a flavoprotein capable of oxidizing β-D-glucose in the presence of oxygen, generating hydrogen peroxide, and gluconic acid, and this process lowers must pH [16]. CAT degrades hydrogen peroxide, generating water and oxygen that can be used again in a new cycle by GOX [17]. Furthermore, the removal of hydrogen peroxide, a highly reactive compound, precludes (i) any inactivation of GOX, (ii) the possibility of any oxidation of other desirable compounds in wine, and (iii) any disruption of the fermentative yeast population [18,19]. 

Research on GOX strategy has been described regarding white grape must (Riesling, Chasselas, [20], Muscat Gordo [21], Muller-Thurgau, Riesling [22,23,24,25,26,27], Rhein Riesling, Pinot Blanc [18] and Riesling [9]) and in red grape must (Pinotage, [28] and Carmenere [29]). These investigations reported differences in glucose oxidation and pH in the musts and alcohol reductions (0.68–4.30% vol. (*v*/*v*)) in the resulting wines as a function of (i) enzyme concentration, (ii) initial must pH, (iii) dissolved oxygen concentration, (iv) agitation, (v) processing time, (vi) temperature, (vii) enzyme origin and purification status, and (viii) grape variety.

Although free GOX–CAT enzymes in the must have been shown to be capable of degrading β-D-glucose and lowering pH with more or less difficulty depending on the variables mentioned, several drawbacks have been reported by these researchers for the optimal application of the GOX–CAT system in winemaking conditions. The main drawback lies in the fact that, to achieve a decrease in alcohol content that compensates for grape over-ripening, there is a significant release of gluconic acid in must, resulting in a pronounced decrease in the must pH. This excessive reduction of pH can inactivate enzymes, modifying the acid–base balances of some compounds and leading to changes in the color and/or sensory characteristics of the wine with a perception of high acidity [18,20,25,26,29]. Furthermore, from the point of view of the smooth running of the enzymatic process, GOX activity is reduced mainly by (i) a low must pH in relation to the optimum pH of the enzyme (5.0–6.0) [30], (ii) the availability of oxygen, and (iii) the temperature [22]. GOX can also be inhibited by hydrogen peroxide if the CAT activity is low or the GOX/CAT ratio used is not optimal [18]. In cases where a must deacidification technique was used to adjust the pH to the optimum pH of GOX, a loss of volatile compounds, increased oxidation, or bad odors in wine can be obtained by the use of chemical deacidifiers or membranes [18,25].

To overcome or minimize these disadvantages, enzyme immobilization has several advantages over the use of free enzymes. Firstly, the immobilization matrix can act as a protective support for enzyme integrity under adverse pH and temperature conditions and thus extend enzyme activity ranges [31,32]. Secondly, the joint immobilization of the GOX and CAT, keeping them close together, can favor the reaction kinetics, such that the hydrogen peroxide generated by GOX will be close to CAT, and the oxygen produced by CAT will be immediately available to GOX [29]. Moreover, it allows for a total control of the reaction time by being able to introduce or remove capsules from the must at will. Finally, immobilized enzymes are reusable, lowering process costs and contributing to a more sustainable process.

Among all immobilization systems described in the scientific literature [33], in situ enzyme entrapment with the formation of a porous matrix is a simple method that is carried out under moderate working conditions while preventing by-products or pH and temperature conditions from degrading the enzyme structure or function. The inclusion prevents the enzymes from diffusing into the must while allowing the transfer of reagents and products. Enzyme activity will depend on the ability of substrates and products to diffuse through the hydrogel, the impact of any internal reagent or product accumulation, and the effective level of oxygen penetration required for GOX activity [34].

Regarding the components of the immobilization material, our group decided to develop a hybrid matrix of an inorganic compound such as silica and an organic one such as alginate [35] to form an interpenetrating polymer network [36]. This silica–calcium–alginate hydrogel was obtained by the aqueous route of the sol–gel process, at room temperature, combining the mechanical strength, low swelling, and resistance to microbial degradation of silica, with the biocompatibility, flexibility, elasticity, and easy handling properties of alginate [37,38]. This silica–calcium–alginate hydrogel was characterized and optimized to entrap the GOX–CAT system [32]. 

This biostrategy is relatively easy to adapt to the winemaking process and is low-cost, eco-friendly, and safe from a food point of view. It is based on the use of the entrapped GOX–CAT to reduce the concentration of β-D-glucose (which accounts for approximately 50% of the fermentable sugars in must) before alcoholic fermentation. Sufficient β-D-glucose oxidation, about 18–36 g/L, leading to a reduction in alcohol content of 1.0–2.0% vol. (*v*/*v*) would compensate for the over-ripening of the grapes [6]. These silica–calcium–alginate hydrogel capsules have been shown to allow for the efficient encapsulation of GOX–CAT, withstand the physico-chemical conditions of the pre-fermentation stage, and enhance the performance of GOX at low temperatures and pH [32]. 

At this point, the study of silica–calcium–alginate hydrogel as an immobilization matrix should focus on verifying its potential to retain the gluconic acid generated by GOX, the main drawback mentioned above. The conventional description of the ionotropic gelation process between sodium alginate and calcium cations involves the widely known “egg-box” model, in which an ionic interaction takes place between calcium cations and guluronic acid monomers of alginate [39]. Moreover, the recovery of gluconic acid during its chemical or biological production involves the utilization of calcium to form calcium gluconate due to the affinity of calcium ions for carboxylate groups present in gluconic acid [40,41]. Therefore, an ionic interaction between the gluconic acid generated by the immobilized GOX and the calcium ions present in the silica–calcium–alginate hydrogel could be expected, resulting in a decreased release of this acid into the must. This partial retention could allow, on the one hand, a moderate and optimal adjustment of the must pH and, on the other hand, would avoid the aforementioned negative effects of high acidity by allowing sufficient oxidation of β-D-glucose to reduce the wine alcohol content by 1.0–2.0% vol. (*v*/*v*). However, the interaction of gluconic acid with calcium cations could potentially reduce the stability of the silica–calcium–alginate hydrogel and its reusability. Therefore, a study on the effectiveness of the co-immobilized enzymes during the incubation time, as well as their operational stability, is necessary.

In this line, the current study aims to verify the hypothesis that this hydrogel can partially retain the gluconic acid by GOX activity, allowing a mild pH decrease without excessive acidification of the must. Moreover, the capacity of the co-immobilized GOX–CAT to reduce the glucose from grape must was tested under enological conditions. Thus, a response surface methodology was carried out to study the concentration of glucose and gluconic acid, the pH, and the color intensity of enzyme-treated musts at different enzyme doses, pH levels, and temperatures. Finally, the efficacy and operational stability of the co-encapsulated enzymes were evaluated.

## 2. Results and Discussion

### 2.1. The Effect of Enzyme Dose, Must pH, and Temperature on the Performance of the Co-Immobilized Enzymes

To examine the performance of co-immobilized enzymes, different enzyme treatments were carried out by varying the dose of co-immobilized GOX–CAT, initial must pH, and incubation temperature under enological conditions (Table 1). Additionally, we aimed to verify the hypothesis that the hydrogel used for GOX–CAT entrapment could partially restrict the release of gluconic acid into the must. Control experiments with the free enzymes were also conducted (Table 2). Glucose consumption, gluconic acid concentration, the yield of gluconic acid from glucose (product–substrate yield), pH decrease, and color intensity increase were examined in the must after the enzyme treatment.

**Figure 1:**
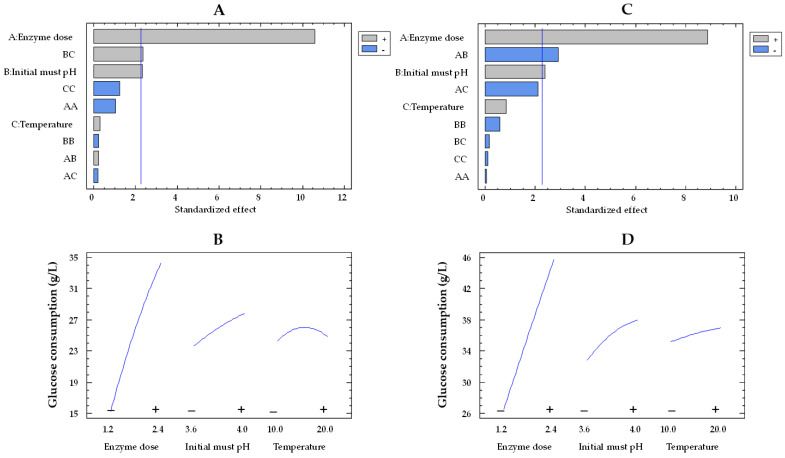
Pareto chart of the standardized effect (**A**) and a main effects plot (−: low; +: high) (**B**) for glucose consumption with the immobilized GOX–CAT system. Pareto chart of the standardized effect (**C**) and a main effects plot (**D**) for glucose consumption with the free GOX–CAT system. Enzyme dose of must is in U/mL; temperature is in °C.

The Pareto plot for glucose consumption by the co-immobilized and free enzymes showed that the effects of enzyme dose and pH were statistically significant, while temperature was not significant (Figure 1A,C, respectively). In this type of chart, statistically significant variables are those that surpass the vertical reference line. The impact of enzyme dose and initial must pH on glucose consumption by immobilized and free enzymes can be observed in Figure 1B,D, respectively. In these plots, the evolution of each variable is depicted from its low level (−) to its high level (+), while the remaining variables are held constant at their mean value. The orientation of the slope (upward or downward) indicates the direction of the effect. A positive slope means that increasing the factor level results in an increase in the response variable, while a negative slope indicates a decrease in the response with increasing factor levels. Moreover, the steepness of the slope shows the magnitude of the effect. A steeper slope means a more significant effect, while a flatter slope suggests a less pronounced impact. As the enzyme dose increased from the lowest to the highest value, the glucose consumption enhanced by a factor of 1.7 (from 26.4 to 45.5 g/L) and 2.2 (from 15.2 to 34.1) for the free and co-immobilized enzymes (Figure 1D,B), respectively, with values estimated at 15 °C and pH 3.8. However, the effect of pH was weaker, as it was observed that, at pH 4.0, the glucose consumption by both free and co-immobilized enzymes increased only by a factor of 1.1 (from 32.6 to 37.4 g/L and from 23.5 to 27.6 g/L, respectively) at an enzyme dose of 1.8 U/mL and 15 °C, compared to pH 3.6. These results agree with those obtained by Ruiz et al. [42] in free and alginate-encapsulated GOX. They also observed that both types of enzymes exhibited a better performance at pH 4.0 compared to pH 3.5. Del Bosque et al. [32] reported the same results for free GOX; however, while the activity of the silica–calcium–alginate immobilized GOX was similar in the pH range of 3–4, the experiments were carried out in glucose solutions and not in must. 

Based on the experiments of Table 1 and Table 2, the mathematical model of Equation (1) (see Section 4.7) enabled the determination of the optimal conditions required to achieve the maximum glucose consumption. For the free enzymes, optimal conditions were found at 2.4 U/mL, pH 3.6, and 10 °C, with a predicted glucose consumption of 48.4 g/L. The optimal conditions for the co-immobilized enzymes (2.4 U/mL, pH 4.0, and 20 °C) were different from those of the free enzymes, resulting in a slightly lower predicted value of 37.8 g/L. These differences in the optimal conditions for both types of enzymes are likely due to the significant effects of the BC (initial must pH and temperature) interaction for immobilized enzymes and the AB (enzyme dose and initial must pH) interaction for free enzymes.

**Figure 2:**
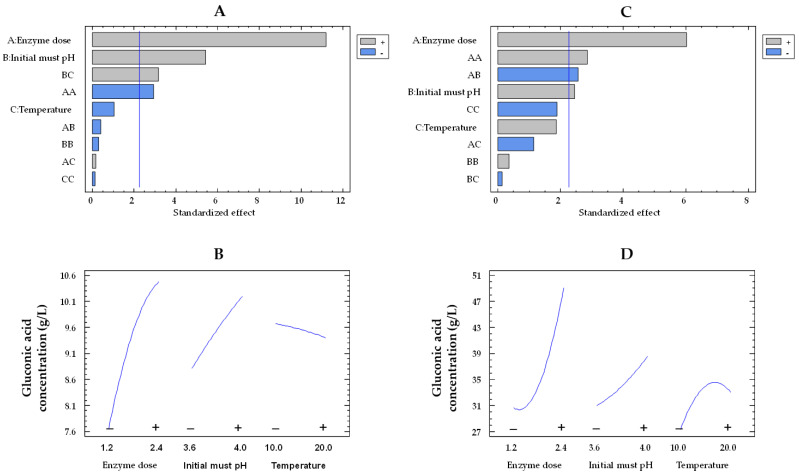
Pareto chart of the standardized effect (**A**) and a main effects plot (−: low; +: high) (**B**) for gluconic acid concentration with the immobilized GOX–CAT system. Pareto chart of the standardized effect (**C**) and a main effects plot (**D**) for gluconic acid concentration with the free GOX–CAT system. Enzyme dose of must is in U/mL; temperature is in °C.

Regarding the concentration of gluconic acid and like glucose consumption, the effects of the enzyme dose and the pH were also statistically significant for both co-immobilized and free enzymes (Figure 2A,C, respectively). Figure 2B,D illustrate the influence of enzyme dose and initial must pH on the concentration of gluconic acid by immobilized and free enzymes, respectively. An increase in the enzyme dose from the lowest to the highest value improved the concentration of gluconic acid in the must by a factor of 1.6 (from 30.7 to 49.2 g/L) and 1.4 (from 7.6 to 10.4 g/L) for the free and co-immobilized enzymes, respectively, at 15 °C and pH 3.8. A 1.2-fold increase in the gluconic acid concentration was observed for both types of enzymes at pH 4.0 compared to pH 3.6 (from 31.1 to 38.6 g/L and from 8.8 to 10.2 g/L, for the free and co-immobilized enzymes, respectively) at 1.8 U/mL and 15 °C.

**Figure 3:**
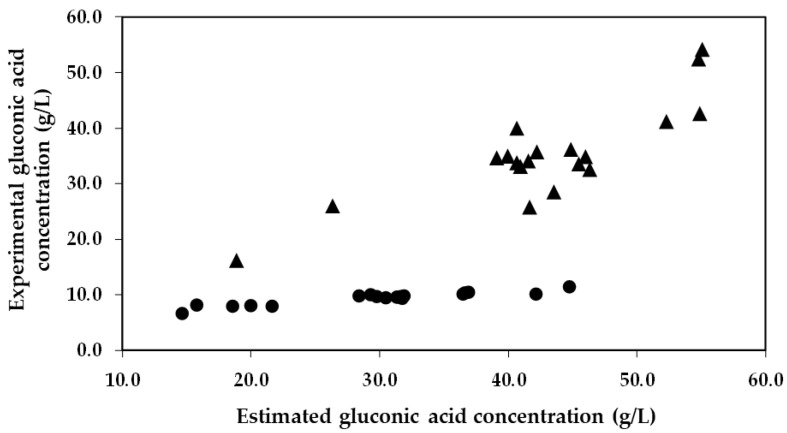
Estimated gluconic acid concentration obtained from glucose oxidation versus the experimental gluconic acid concentration obtained in the must for each run of the Box–Behnken design. The circles (●) represent the data of the immobilized GOX–CAT system, and the triangles (▲) represent the data of the free enzymes.

It is of particular importance to emphasize that the concentration of gluconic acid found in the encapsulated enzyme-treated musts was significantly lower than what was expected. This trend can be visualized in Figure 3, which presents a comparison between the estimated concentration of gluconic acid (calculations based on the glucose consumptions) and the experimental concentration of gluconic acid obtained from the 19 trials. As an example, in Trial 6 and 17 with co-immobilized enzymes, experiments characterized by a low and high glucose consumption, an estimated production of gluconic acid of 18.6 and 44.7 g/L was estimated, respectively. However, significantly lower concentrations of gluconic acid were detected in the musts (6.5 and 11.3 g/L, respectively), representing 34.9% and 25.3% of the estimated gluconic acid produced by GOX. Nevertheless, in the same trials (6 and 17) with free enzymes, the estimated production (26.3 and 55.1 g/L, respectively) closely matched the concentrations detected in the musts (25.9 and 54.1 g/L, respectively), indicating that 98.5% and 98.2% of the estimated gluconic acid produced by the free GOX was detected in the musts, respectively. 

**Figure 4:**
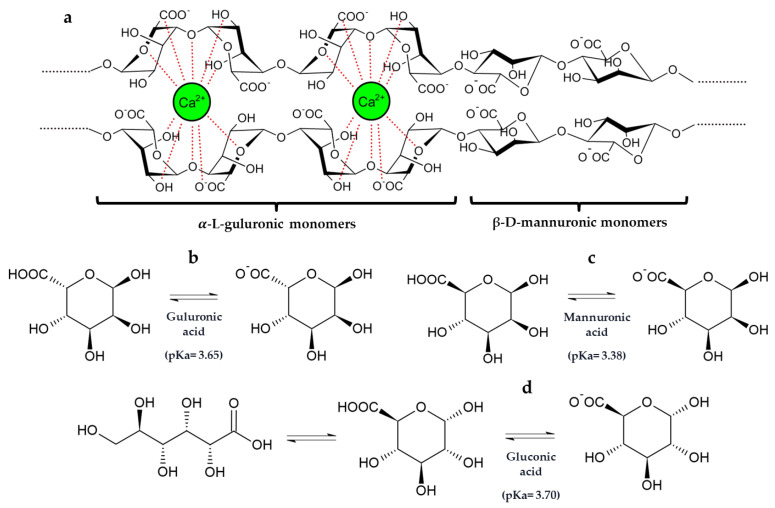
(**a**) Two-chain fragment of the alginate polymeric structure consisting of α-L-guluronic and β-D-mannuronic acids monomers and their interactions with calcium ion according to the “egg-box model”. Stereo projections and pKa of α-L-guluronic (**b**) and β-D-mannuronic (**c**) acids present in sodium alginate and gluconic acid (**d**) [44,45].

These results highlight that the co-immobilization of GOX–CAT in silica–calcium–alginate gels reduced the amount of gluconic acid released into the must, probably due to an interaction of gluconic acid with the calcium of the capsule structure. In fact, gluconic acid has the capacity to chelate cations such as calcium [43]. Furthermore, it is also noteworthy that the pK_a_ values and the chemical structure of gluconic acid are very similar to those of mannuronic and guluronic acids from the alginate structure (Figure 4) [44,45]. 

**Figure 5:**
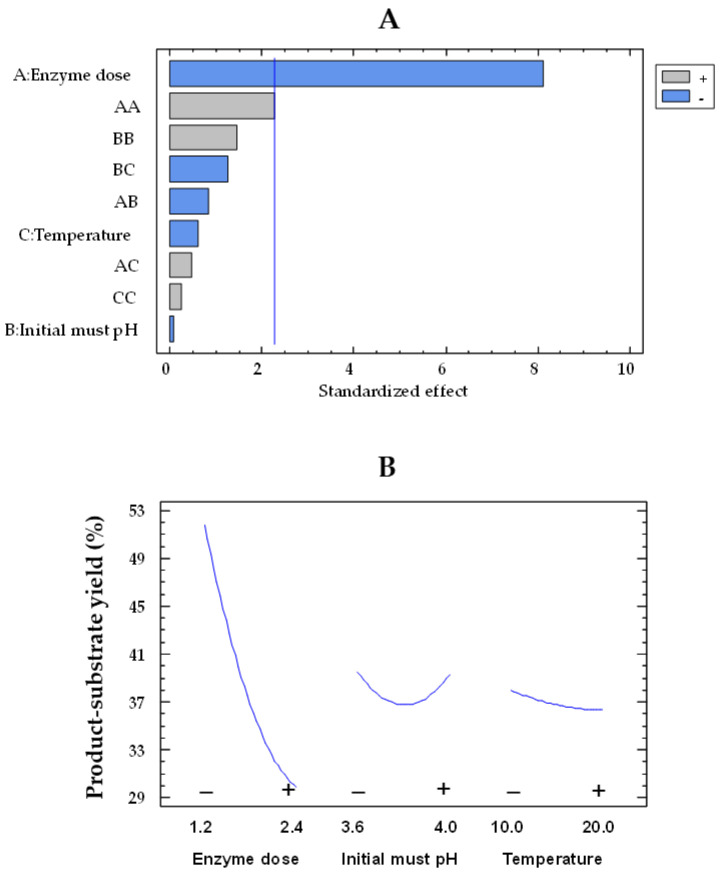
Pareto chart of the standardized effect (**A**) and a main effects plot (−: low; +: high) (**B**) for the product–substrate yield (%) with the immobilized GOX–CAT system. Enzyme dose of must is in U/mL; temperature is in °C.

At this point, it is interesting to identify the factors and their levels that minimize the product–substrate yield. For free enzymes, an average product–substrate yield of 99.5% was obtained. However, the selected model for the product–substrate yield (Equation (1)) was inadequate to describe the observed data from free GOX-treated musts, as the *p*-value for the test of lack of fit was <0.05, indicating that the studied variables had a limited influence on the yield of free enzymes. However, the Pareto plot for co-immobilized enzymes showed that the enzyme dose effect was negative and statistically significant (Figure 5A). 

**Figure 6:**
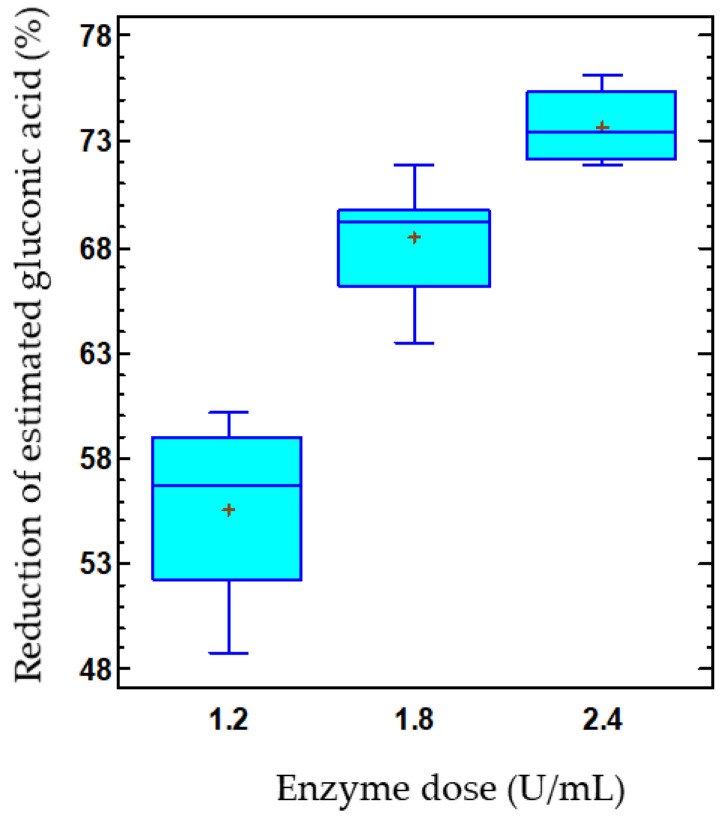
Effect of the co-immobilized enzyme dose on the reduction of estimated gluconic acid (%) in must. The data is represented using box-and-whisker plots, where the lower and upper edges of the box represent the first and third quartiles, respectively. The middle line within the box always represents the second quartile, also known as the median. The vertical lines extending from the boxes, known as whiskers, indicate the highest and lowest values in the dataset. Additionally, plus signs (+) are used to indicate the mean for each group.

The product–substrate yield by immobilized enzymes in response to enzyme dose can be observed in Figure 5B. Thus, the yield decreased as the dose of co-immobilized enzymes increased. At the lowest enzyme dose, also corresponding to the lowest capsule concentration, a yield of 51.8% was achieved, which decreased to 29.9% at the highest concentration, with values estimated at 15 °C and pH 3.8. Likewise, a reduction of 55.6% in the estimated gluconic acid was found in the musts treated with the lowest capsule concentration, while this reduction increased to 73.7% for the highest capsule concentration (Figure 6). Thus, the release of gluconic acid from the capsules was reduced as the concentration of capsules used increased, even though the glucose consumption and, consequently, the gluconic acid production also enhanced. These results are consistent with those previously discussed, indicating a possible accumulation of gluconic acid in the capsules.

**Figure 7:**
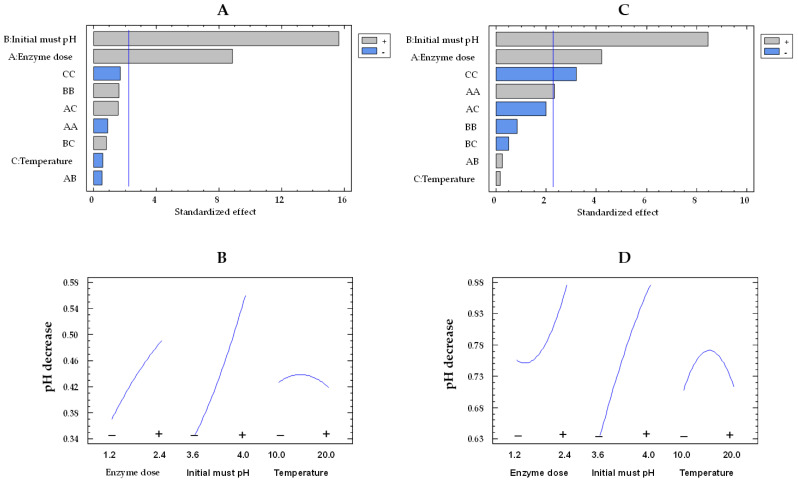
Pareto chart of the standardized effect (**A**) and a main effects plot (−: low; +: high) (**B**) for pH decrease with the immobilized GOX–CAT system. Pareto chart of the standardized effect (**C**) and a main effects plot (**D**) for pH decrease with the free GOX–CAT system. Enzyme dose of must is in U/mL; temperature is in °C.

In relation to pH decrease, the musts treated with the free enzymes exhibited higher values than those treated with the co-immobilized enzymes, consistent with the results observed for glucose consumption and gluconic acid concentration. As can be observed in the Pareto chart (Figure 7A,C), initial pH and enzyme dose effects were statistically significant for the co-immobilized and free enzymes. Examining Figure 7B,D allows for an observation of the impact of enzyme dose and initial must pH on pH decrease by both immobilized and free enzymes, respectively. On the one hand, the pH decrease was higher at pH 4.0 than at pH 3.6, as a result of an enhanced production of gluconic acid at pH 4.0 [46]. As the initial must pH increased from the lowest to the highest value, the pH decrease enhanced by a factor of 1.3 (from 0.64 to 0.86 units) and 1.6 (from 0.34 to 0.56 units) for the free and co-immobilized enzymes, respectively, at 15 °C and 1.8 U/mL. On the other hand, as the enzyme dose rose, there was an increment in the pH decrease. This rise was by a factor of 1.1 (from 0.75 to 0.86 units) and 1.3 (from 0.37 to 0.49 units) for the free and co-immobilized enzymes, respectively.

**Figure 8:**
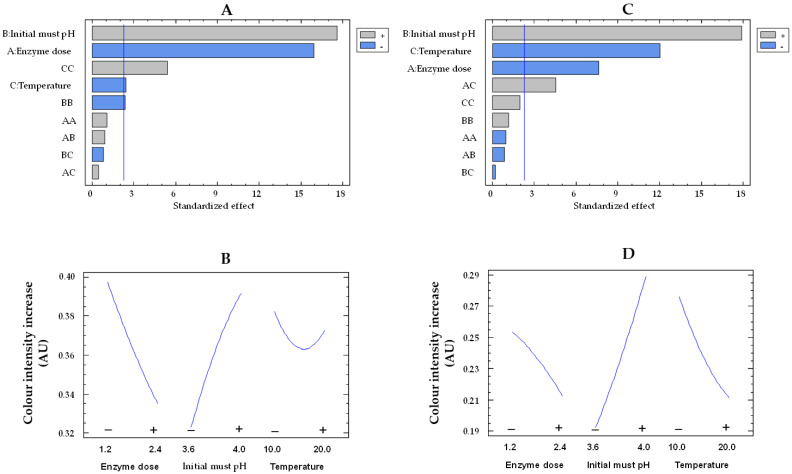
Pareto chart of the standardized effect (**A**) and a main effects plot (−: low; +: high) (**B**) for color intensity increase with the immobilized GOX–CAT system. Pareto chart of the standardized effect (**C**) and a main effects plot (**D**) for color intensity increase with the free GOX–CAT system. Enzyme dose of must is in U/mL; temperature is in °C.

The must treatment with the free and co-immobilized enzymes caused an increase in the color intensity of the musts, likely as a result of the conversion of phenols to quinones by polyphenoloxidases, which subsequently polymerize to form macromolecules with a yellow-brown color [47]. The Pareto chart indicates that the three studied variables are statistically significant for both types of enzymes (Figure 8A,C). Figure 8B,D display how enzyme dose, initial must pH, and temperature influence color intensity increase by immobilized and free enzymes, respectively. With increasing pH from 3.6 to 4.0, there was a 1.5-fold (from 0.19 to 0.29 AU) and 1.2-fold (from 0.32 to 0.39 AU) rise in the color intensity increase for the free and co-immobilized enzymes, respectively. The enzymatic oxidation of phenols in white must has been reported to be more pronounced at higher pH levels [48]. 

In addition, by increasing the enzyme dose from the lowest to the highest values, there was a 1.2-fold drop in color intensity increase for both free enzymes (from 0.25 to 0.21 AU) and co-immobilized enzymes (from 0.39 to 0.33 AU). This finding could be produced by at least two causes. First, as the concentration of both free and co-immobilized enzymes increased, so did the glucose consumption and the must acidification, thus resulting in a reduction of the must oxidation at low pH [48]. Second, an adsorption of phenols or their oxidation products on the surface of the silica–calcium–alginate capsules could take place [49,50], leading to a reduction in the color intensity increase of the must. Finally, the temperature rise reduced the color intensity increase in both the free and co-immobilized enzyme-treated musts. These results could be due to a lower oxidation of phenols by the acidification of the must, as a higher consumption of glucose was observed with increasing temperature, although without statistically significant differences. 

From an enological point of view, a viable biotechnological approach to achieve a moderate reduction in the potential alcohol strength of the must (e.g., 1–2% vol. (*v*/*v*)), along with low levels of gluconic acid and minimal color intensity increase, could involve must treatment with a high dose of co-immobilized enzymes. The enzyme reaction could be stopped once the desired potential alcohol strength of the must is reached by removing the immobilized enzymes by filtration, likely within a reaction time of less than 24 h. Another potential strategy could be to employ a high concentration of capsules over a period of 48 h, resulting in an excessive reduction in the potential alcohol content of the must. This reduction can then be rectified by blending the enzyme-treated must with untreated must in the appropriate proportion to achieve the desired reduction in potential alcohol content. The latter strategy could avoid an excessive aeration of the entire must volume [51].

### 2.2. Efficiency of the Co-Immobilized and Free GOX

**Figure 9:**
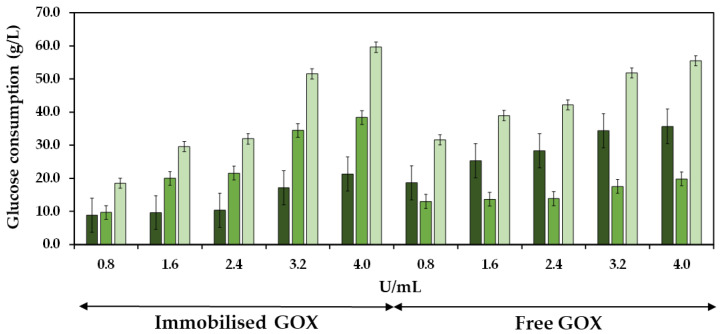
Glucose consumption is shown as a function of GOX units per mL of must, with both immobilized and free enzymes, during the first 24 h (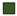), the second 24 h (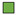), and the total accumulated consumption in the 48 h (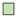) of pre-fermentative treatment. Bars represent the 95% confidence interval.

The efficiency of the co-immobilized and free GOX was studied by analyzing the consumption of glucose during both the initial and the subsequent 24 h periods of must treatment at different enzyme doses. Figure 9 shows the glucose consumption during the first and second 24 h periods of enzyme reaction, as well as the total glucose consumption at 48 h at different doses of the co-immobilized enzymes. The experiments were also performed with the free enzymes for control purposes. The total glucose consumption after 48 h of enzyme treatment increased as the enzyme dose increased, reaching 55.5 and 59.6 g/L at 4.0 U/mL of free and co-immobilized enzymes, respectively, resulting in a notable reduction of the potential alcoholic strength of must at 3.2 and 3.5% vol. (*v*/*v*), respectively. Regarding the free enzymes, the most significant glucose consumption occurred within the initial 24 h period, followed by a subsequent decline in glucose consumption. In contrast, the behavior of the co-immobilized enzymes was different, as the glucose consumption during the initial 24 h period was lower compared to the consumption observed during the subsequent 24 h period. In the initial 24 h period, the ratio of glucose consumed per enzyme dose was 52.1% higher for the free enzymes (0.98 g/U) compared to the co-immobilized enzymes (0.64 g/U). However, in the subsequent 24 h period, the ratio was 87.1% higher for the co-immobilized enzymes (1.63 g/U) compared to the free ones (0.21 g/U), indicating an increased efficiency of the co-immobilized enzymes along the reaction time [52].

### 2.3. Operational Stability of the Co-Immobilized GOX

**Figure 10:**
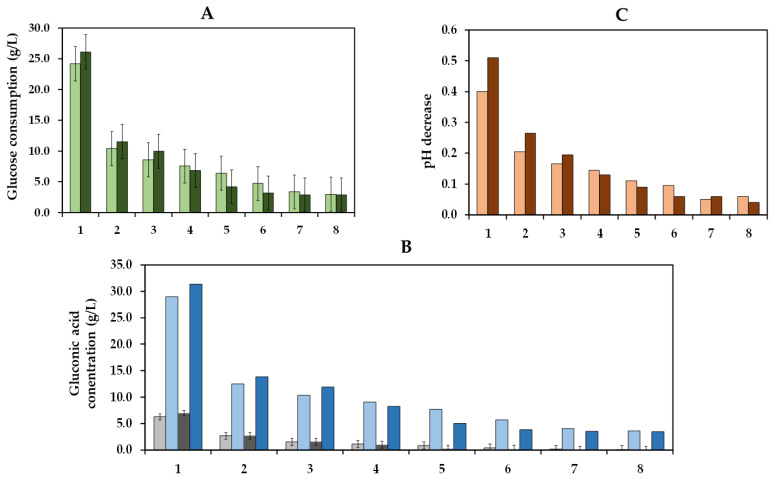
Glucose consumption (g/L) at initial must pH levels of 3.8 (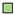) and 4.0 (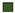) is plotted as a function of cycles of 48 h each (**A**). Experimental (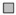) and estimated (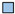) gluconic acid concentration (g/L) at an initial must pH of 3.8 and experimental (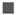) and estimated (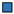) gluconic acid concentration (g/L) at an initial must pH of 4.0 are plotted as a function of enzyme treatment cycles of 48 h each (**B**). The pH decrease produced in the must at initial pH levels of 3.8 (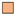) and 4.0 (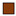) is shown as a function of the enzyme treatment cycles (**C**). Bars, in all graphs, represent the 95% confidence interval.

The operational stability is a crucial factor that determines the practical and economic viability of an immobilized enzyme system in industrial applications [53,54]. Thus, we studied the ability of the co-immobilized enzymes to maintain their enzyme activity over eight cycles of use, which involved the repetitive utilization of these enzymes in eight reaction cycles (Figure 10). In the first cycle, the glucose consumption at pH 3.8 (24.2 g/L) was similar to that at pH 4.0 (26.1 g/L). Subsequently, a significant reduction in glucose consumption was observed in consecutive cycles, achieving a glucose consumption of 10.4 and 11.5 g/L in the second cycle and only of 3.0 and 2.9 g/L in the last cycle at pH 3.8 and pH 4.0, respectively. Comparable performance was obtained for gluconic acid, with concentrations of 6.3 and 7.0 g/L during the first cycle and of only 2.6 and 2.7 g/L during the second cycle at pH 3.8 and pH 4.0, respectively. In terms of pH, reductions of 0.40 and 0.51 units in the first cycle and of 0.21 and 0.27 units in the second cycle were observed at pH 3.8 and pH 4.0, respectively. The drop in pH decrease can be attributed to a lower glucose consumption due to the recycling of the co-immobilized enzymes.

In contrast to the results shown by Del Bosque et al. [32], where the co-immobilized GOX–CAT were successfully reused for eight cycles of 90 min in a glucose solution, in this assay with Verdejo must, a decrease in glucose consumption was observed due to the reuse of the co-immobilized enzymes. These differences may be attributed to several factors. Initially, the build-up of macromolecules, including proteins, polysaccharides, and polyphenols from the must, within the pores of the capsules could hinder the movement of substrates and products. These macromolecules could also potentially interact with the active center of the enzymes, hindering the ability of enzymes to interact effectively with their respective substrates. Moreover, the higher duration of each cycle (48 h) could enhance the accumulation of gluconic acid and hydrogen peroxide inside the capsules, inhibiting the activity of GOX [19,55]. Additionally, enzyme release from the hydrogel is also possible, potentially enhanced by a destabilization of the hydrogel caused by the interaction between gluconic acid and calcium cations [56].

## 3. Conclusions

The results of this study demonstrated that silica–calcium–alginate hydrogel with co-immobilized GOX–CAT exhibited a high capacity to simultaneously decrease the glucose content and reduce the release of gluconic acid in must. Below are the specific conclusions:

− A noteworthy glucose consumption of up to 37.3 g/L was observed with co-immobilized enzymes, resulting in a reduction in the potential alcoholic strength of the must by about 2.0% vol. (*v*/*v*).
− A remarkable reduction of up to 73.7% in the estimated gluconic acid concentration was achieved in the co-immobilized enzyme-treated musts, mitigating the risk of an excessive must acidity observed with free enzymes.
− Higher enzyme doses enhanced the pH decrease of must, observing a pH decrease of up to 1.02 with free enzymes and only up to 0.60 with co-immobilized enzymes.
− The rise in the color intensity of the must became less pronounced as the dose of co-immobilized enzymes increased (from 0.39 to 0.33 AU).
− A gradual decline in glucose consumption was observed over eight consecutive cycles of use of the co-immobilized enzymes.

Overall, our findings provide valuable insights into the performance of co-immobilized GOX–CAT for must treatment to produce reduced-alcohol wines. In terms of the glucose consumption, gluconic acid release, and color intensity of the must, the use of co-immobilized GOX–CAT is very promising. However, their application in winemaking requires further studies focusing on oxygen management, as well as on how to apply the co-immobilized enzymes in the winemaking process and their impact on the physico-chemical and sensory quality of the wine.

## 4. Materials and Methods

### 4.1. Enzymes and Chemical Reagents

GOX (EC 1.1.3.4, Gluzyme^®^ Fortis 10,000 BG from *Aspergillus niger*, 10,000 U/g) and CAT (EC 1.11.1.6, Catazyme^®^ 25 L from *A. niger*, 25,000 U/mL) were kindly provided by Novozymes (Bagsvaerd, Denmark). LUDOX^®^ HS-40 colloidal silica (420816) and sodium silicate (338443) were purchased from Sigma-Aldrich (St. Louis, MO, USA). Sodium alginate (A3249,0250) was acquired from Panreac Applichem (Darmstadt, Germany). The remaining chemicals were of analytical grade and were obtained from Panreac, S.A. (Madrid, Spain).

### 4.2. Verdejo Grape Must

For all assays, Verdejo grapes harvested in 2020 from a winery located in the Appellation of Origin Rueda were used. The grapes were destemmed and crushed in a crusher-destemmer machine equipped with a horizontal roller (Model Europa 2, FS group, Castellón de la Plana, Spain) and pressed in a vertical hydraulic press of 40 L (WilTec Wildanger Technik GmbH, Eschweiler, Germany) at a pressure of 0.2 MPa. The obtained must was then kept frozen at −20 °C in an industrial freezing chamber until its use. Prior to the assays, the must was thawed at room temperature and homogenized at 250 rpm (Orbital Shaker SO1, Stuart Scientific, Stone, UK) for 20 min. The must was then centrifuged at 2320× *g* for 5 min (Sorvall ST 8R Centrifuge, Osterode am Harz, Germany) to remove plant materials and precipitates that could interfere with the enzyme reaction. The basic composition of the must was the following: 21.9 ± 0.1 °Brix; pH 3.59 ± 0.01; 4.3 ± 0.1 g/L of total acidity expressed as tartaric acid; 30 ± 2 mg/L of free SO_2_; 50 ± 5 mg/L of total SO_2_. The must was analyzed according to OIV methods [57]. The must was adjusted to different pH values (3.6–3.8–4.0) by adding 4 M NaOH (Section 4.4 and Section 4.5).

### 4.3. Co-Immobilization of GOX and CAT in Silica–Calcium–Alginate Capsules

**Figure 11:**
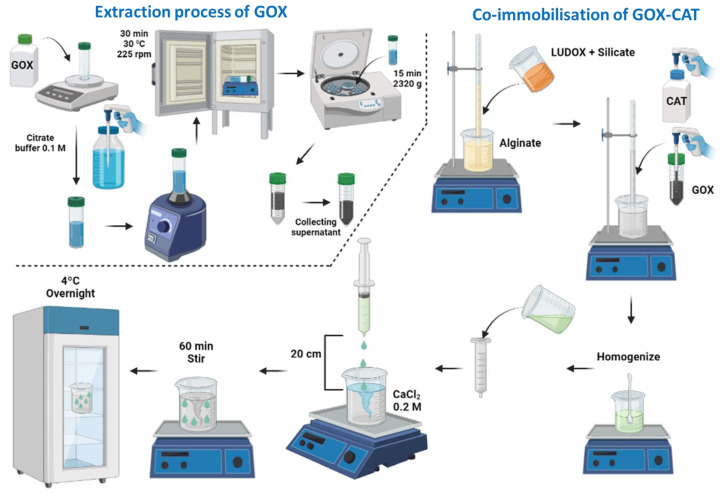
Flow chart of the extraction process of the GOX enzyme from its floury matrix and the general procedure to obtain the silica–calcium–alginate capsules with the immobilized GOX–CAT enzymes.

The co-immobilization of GOX and CAT was carried out using the Interpenetrated Polymer Network Method by entrapment in mixed silica–calcium–alginate hydrogels. This method is based on the mixture of silicon derivatives with sodium alginate prior to gelation in the presence of calcium (Figure 11). The co-immobilization of GOX and CAT was performed at the optimum values and conditions indicated by Del-Bosque et al. [32]. 

Gluzyme Fortis 10,000, which is a formulation used in the baking industry with wheat flour as a matrix, was not directly employed. To prepare the GOX extract, 3 g of Gluzyme was dissolved in 100 mL of 0.1 M citrate buffer at pH 6.6. The resulting mixture was stirred for 30 min and then subjected to centrifugation at 2320× *g* for 15 min using a Sorvall ST 8R Centrifuge [29] (Figure 11). The supernatant obtained was used as a source of GOX. Catazyme (25 L) was directly used.

### 4.4. Must Treatment with Co-Immobilized Enzymes 

Response Surface Methodology was used to evaluate and compare the co-immobilized and free enzyme activity at three different variables with respect to five dependent variables (responses). Thus, two trials in grape must with (a) GOX–CAT co-immobilized in silica–calcium–alginate capsules and (b) free enzymes were carried out simultaneously and under the same experimental conditions. The statistical significance of the three independent variables—(i) an enzyme dose of co-immobilized GOX–CAT (1.2–1.8–2.4 U/mL of must for both enzymes, corresponding to 38–57–76 mg of gel/mL of must) or free GOX–CAT (1.2–1.8–2.4 U/mL of must for both enzymes), (ii) initial must pH (3.6–3.8–4.0), and (iii) temperature of incubation (10–15–20 °C)—was evaluated in relation to five dependent response variables—(I) glucose consumption (g/L), (II) gluconic acid concentration (g/L), (III) yield of gluconic acid from glucose (product–substrate yield) (%), (IV) pH decrease, and (V) color intensity increase (AU). For this purpose, a Box–Behnken experimental design, rotatable, orthogonal, and quadratic processed, with 7 central points for the estimation of the experimental error, was chosen, generating 19 experiments (Table 1 and Table 2). All experiments were carried out under agitation at 150 rpm (Orbital Shaker SO1) in aliquots of 5 mL of must.

The initial and final concentration of both glucose and gluconic acid were measured with enzyme kits (K-FRGLQR-02/17 and K-GATE 04/20, respectively, Megazyme Bray Co., Wicklow, Ireland). Three aliquots of each type of must (Run 1–19) were taken, and each aliquot was measured in duplicate. The estimated gluconic acid production (g/L) was calculated from the glucose consumption as a 1:1 molar equivalence. The initial and final pH values of the must were measured with a pHmeter (sensION^TM^+ HACH-LANGE, Barcelona, Spain) and the initial and final color intensity was determined at 420 nm using a UV-Vis spectrophotometer (Genesys™ 150 Vis/UV-Vis, Thermo Fisher, Madrid, Spain). For both determinations, three aliquots of each type of must (Run 1–19) were taken and measured.

### 4.5. Efficiency of Co-Immobilized GOX over the Reaction Time

The glucose consumption in the must was determined at the first and second 24 h period of enzyme reaction in 5 mL of Verdejo grape must at pH 3.6. The must was incubated at five different doses of co-immobilized enzymes (0.8, 1.6, 2.4, 3.2, and 4.0 U/mL of must for both GOX and CAT enzymes, corresponding to 25, 51, 76, 101, and 127 mg of gel/mL of must). With the same doses and experimental conditions, the free enzymes were added directly into the must as controls. All assays were carried out simultaneously in triplicate at 15 °C and 150 rpm (Orbital Shaker SO1). Aliquots of enzyme-untreated musts were also used as controls. Glucose consumption (g/L) was determined in duplicated following the method of Section 4.4.

### 4.6. Operational Stability of Co-Immobilized Enzymes

The activity of the co-immobilized enzymes was examined over eight cycles of reuse of 48 h in 5 mL of must with the initial pH adjusted to 3.8 and 4.0 and in triplicate. An enzyme dose of 1.6 U/mL of must (51 mg gel/mL must) for both GOX and CAT was used. The capsules were washed after each cycle with 30 mL of CaCl_2_ for 30 s and 30 mL of distilled water for 30 s consecutively before starting the next cycle. All assays were performed at 15 °C and 150 rpm. In each cycle, enzyme-untreated musts were used as controls. Glucose consumption (g/L) and gluconic acid concentration (g/L) were measured in triplicate during each cycle, while pH was measured once, following the methods described in Section 4.4.

### 4.7. Statistical Analysis

Analysis of the experimental design of the experimental data was performed at a significant level of α = 0.05 using Statgraphics Centurion 19 (version 19.2.01, The Plains, VA, USA). The significance of the effects of each variable were established fixing a second-order model for the independent variables with a significance level (α) of 0.05 and 10 coefficients as shown in Equation (1) [58].

(1) $${y=\beta }_{o}+\sum_{i=1}^{k}\beta_{i}X_{i}+\sum_{i=1}^{k}{\beta_{ii}X}_{i}^{2}+\sum_{i}^{i<j}\;\;\sum_{j}\beta_{ij}X_{i}X_{j}\;\;+\varepsilon$$

where *y* is a dependent response variable, $X_{i}$ and $X_{j}\;$ are the three independent factors, $\beta_{o}$, $\beta_{i}$, $\beta_{ii}$, and $\beta_{ij}$ are regression coefficients, and ε is the error. The outcome of the ANOVA can be visualized in a Pareto plot, where the absolute value of the standardized estimated effect of each factor investigated on a dependent response variable is plotted.

## Figures and Tables

**Table 1:** Box–Behnken design with 3 experimental factors and 19 runs to evaluate the five response variables with the immobilized GOX–CAT system.

	Experimental Factors			Response Variables ^1^				
Run	Enzyme Dose (U/mL)	Initial Must pH	Temperature (°C)	Glucose Consumption (g/L)	Gluconic Acid Concentration (g/L)	pH Decrease	Color Intensity Increase (AU)	Product–Substrate Yield (%)
1	1.8	4.0	20.0	30.79 ± 1.72	10.36 ± 0.39	0.55 ± 0.01	0.406 ± 0.001	33.64 ± 3.08
2	1.2	4.0	15.0	13.17 ± 1.71	8.10 ± 0.39	0.51 ± 0.01	0.418 ± 0.001	61.50 ± 10.85
3	2.4	3.8	20.0	30.56 ± 1.72	10.28 ± 0.39	0.49 ± 0.01	0.340 ± 0.001	33.63 ± 3.10
4	1.8	3.8	15.0	26.42 ± 1.72	9.60 ± 0.39	0.47 ± 0.01	0.363 ± 0.001	36.32 ± 3.76
5	1.8	3.6	20.0	18.08 ± 1.72	7.92 ± 0.39	0.33 ± 0.01	0.335 ± 0.001	43.82 ± 6.24
6	1.2	3.8	10.0	15.50 ± 1.71	7.87 ± 0.39	0.37 ± 0.01	0.423 ± 0.001	50.76 ± 8.03
7	1.8	3.6	10.0	23.70 ± 1.72	9.74 ± 0.39	0.34 ± 0.01	0.332 ± 0.001	41.08 ± 4.54
8	1.8	3.8	15.0	26.12 ± 1.72	9.55 ± 0.39	0.43 ± 0.01	0.367 ± 0.001	36.55 ± 3.82
9	1.8	3.8	15.0	24.83 ± 1.72	9.61 ± 0.39	0.44 ± 0.01	0.365 ± 0.001	38.69 ± 4.16
10	2.4	3.8	10.0	30.42 ± 1.72	10.06 ± 0.39	0.48 ± 0.01	0.355 ± 0.001	33.06 ± 3.08
11	1.8	3.8	15.0	26.48 ± 1.72	9.35 ± 0.39	0.43 ± 0.01	0.360 ± 0.001	35.31 ± 3.69
12	1.2	3.6	15.0	12.24 ± 1.72	6.54 ± 0.40	0.28 ± 0.01	0.361 ± 0.001	53.39 ± 10.61
13	2.4	3.6	15.0	35.16 ± 1.72	10.07 ± 0.39	0.39 ± 0.01	0.297 ± 0.001	28.64 ± 2.45
14	1.2	3.8	20.0	16.68 ± 1.71	7.97 ± 0.39	0.32 ± 0.01	0.403 ± 0.001	47.76 ± 7.16
15	1.8	3.8	15.0	26.22 ± 1.72	9.58 ± 0.39	0.44 ± 0.01	0.365 ± 0.001	36.55 ± 3.80
16	1.8	4.0	10.0	24.46 ± 1.72	9.93 ± 0.39	0.53 ± 0.01	0.412 ± 0.001	40.58 ± 4.36
17	2.4	4.0	15.0	37.30 ± 1.72	11.34 ± 0.39	0.60 ± 0.01	0.364 ± 0.001	30.40 ± 2.39
18	1.8	3.8	15.0	25.43 ± 1.72	9.41 ± 0.39	0.41 ± 0.01	0.362 ± 0.001	37.00 ± 3.95
19	1.8	3.8	15.0	26.60 ± 1.72	9.80 ± 0.39	0.45 ± 0.01	0.362 ± 0.001	36.84 ± 3.77

^1^ Glucose consumption and gluconic acid concentration are grams of β-D-glucose consumed per L of must and g of gluconic acid per L of must, respectively, to assess the activity of the GOX–CAT system. pH decrease is the acidification of the must by gluconic acid after enzyme treatment compared to the initial pH. Color intensity increase is the change in absorbance at 420 nm measured in the must after enzyme treatment compared to the initial absorbance. Product–substrate yield (%) is the percentage of the quotient between gluconic acid concentration and glucose consumption. Each value represents the mean ± its 95% confidence interval.

**Table 2:** Box–Behnken design with 3 experimental factors and 19 runs to evaluate the five response variables with the free enzyme GOX–CAT system.

	Experimental Factors			Response Variables ^1^				
Run	Enzyme Dose (U/mL)	Initial Must pH	Temperature (°C)	Glucose Consumption (g/L)	Gluconic Acid Concentration (g/L)	pH Decrease	Color Intensity Increase (AU)	Product–Substrate Yield (%)
1	1.8	4.0	20.0	35.18 ± 1.72	35.64 ± 0.69	0.78 ± 0.01	0.262 ± 0.001	101.30 ± 6.83
2	1.2	4.0	15.0	33.88 ± 1.72	39.88 ± 0.77	0.86 ± 0.01	0.311 ± 0.001	117.69 ± 8.16
3	2.4	3.8	20.0	43.60 ± 1.72	41.14 ± 0.80	0.77 ± 0.01	0.205 ± 0.001	94.36 ± 5.48
4	1.8	3.8	15.0	37.42 ± 1.72	36.12 ± 0.70	0.84 ± 0.01	0.219 ± 0.001	96.54 ± 6.22
5	1.8	3.6	20.0	34.63 ± 1.72	34.01 ± 0.66	0.60 ± 0.01	0.169 ± 0.001	98.20 ± 6.70
6	1.2	3.8	10.0	21.95 ± 1.72	25.93 ± 0.52	0.66 ± 0.01	0.310 ± 0.001	118.12 ± 11.46
7	1.8	3.6	10.0	34.73 ± 1.72	25.69 ± 0.52	0.59 ± 0.01	0.232 ± 0.001	73.98 ± 5.11
8	1.8	3.8	15.0	33.29 ± 1.72	34.91 ± 0.68	0.78 ± 0.01	0.235 ± 0.001	104.85 ± 7.35
9	1.8	3.8	15.0	38.36 ± 1.72	34.77 ± 0.67	0.79 ± 0.01	0.247 ± 0.001	90.66 ± 5.74
10	2.4	3.8	10.0	45.75 ± 1.72	42.48 ± 0.83	0.83 ± 0.01	0.236 ± 0.001	92.85 ± 5.23
11	1.8	3.8	15.0	38.62 ± 1.72	32.45 ± 0.63	0.73 ± 0.01	0.245 ± 0.001	84.02 ± 5.30
12	1.2	3.6	15.0	15.72 ± 1.72	16.04 ± 0.41	0.59 ± 0.01	0.206 ± 0.001	102.08 ± 13.63
13	2.4	3.6	15.0	45.67 ± 1.73	52.39 ± 1.04	0.73 ± 0.01	0.170 ± 0.001	114.72 ± 6.51
14	1.2	3.8	20.0	32.60 ± 1.72	34.55 ± 0.67	0.76 ± 0.01	0.210 ± 0.001	105.98 ± 7.54
15	1.8	3.8	15.0	37.90 ± 1.72	33.42 ± 0.65	0.73 ± 0.01	0.240 ± 0.001	88.19 ± 5.63
16	1.8	4.0	10.0	36.31 ± 1.72	28.46 ± 0.56	0.81 ± 0.01	0.328 ± 0.001	78.38 ± 5.19
17	2.4	4.0	15.0	45.92 ± 1.73	54.11 ± 1.08	1.02 ± 0.01	0.262 ± 0.001	117.83 ± 6.67
18	1.8	3.8	15.0	34.15 ± 1.72	32.98 ± 0.64	0.75 ± 0.01	0.234 ± 0.001	96.58 ± 6.65
19	1.8	3.8	15.0	33.91 ± 1.72	33.62 ± 0.65	0.78 ± 0.01	0.236 ± 0.001	99.15 ± 6.86

^1^ Glucose consumption and gluconic acid concentration are grams of β-D-glucose consumed per L of must and g of gluconic acid per L of must, respectively to assess the activity of GOX–CAT system. pH decrease is the acidification of the must by gluconic acid after enzyme treatment compared to the initial pH. Color intensity increase is the change in absorbance at 420 nm measured in the must after enzyme treatment compared to the initial absorbance. Product–substrate yield (%) is the percentage of the quotient between gluconic acid concentration and glucose consumption. Each value represents the mean ± its 95% confidence interval.

## Data Availability

Not applicable.
